# Barriers in household access to medicines for chronic conditions in three Latin American countries

**DOI:** 10.1186/s12939-015-0254-z

**Published:** 2015-10-31

**Authors:** Isabel Cristina Martins Emmerick, Vera Lucia Luiza, Luiz Antonio Bastos Camacho, Catherine Vialle-Valentin, Dennis Ross-Degnan

**Affiliations:** Department of Population Medicine, Harvard Medical School and Harvard Pilgrim Health Care Institute, 133 Brookline Avenue, 6th Floor, Boston, MA 02215 USA; Center for Pharmaceutical Policies, Sergio Arouca National School of Public Health, Oswaldo Cruz Foundation, (NAF/ENSP/Fiocruz), Rua Leopoldo Bulhões 1480, Manguinhos, 21041 210 Rio de Janeiro, RJ Brazil; Department of Epidemiology and Quantitative Methods in Health, Sergio Arouca National School of Public Health, Oswaldo Cruz Foundation (DEMEQS/ENSP/Fiocruz), Rua Leopoldo Bulhões 1480, Manguinhos, 21041 210 Rio de Janeiro, RJ Brazil

**Keywords:** Chronic disease, Central America, Household, Health services accessibility, Health systems

## Abstract

**Background:**

Access to medicines is one of the major challenges in health policy. The high out-of-pocket expenditures on medicines in the Latin American and Caribbean (LAC) region represents important barrier to affordable access to care for NCDs. This paper aim to identify key barriers in access to medicines for household members with a diagnosed chronic condition in three Central America countries.

**Methods:**

This was a cross-sectional analytic study, based on data from three household surveys using a common methodology. We examined associated factors to: (1) seeking care for chronic illness from a trained clinician in the formal health system, and (2) obtaining all medicines sought for the chronic conditions reported.

**Results:**

A chronic condition was reported in 29.8 % (827) of 2761 households - 47.0, 30.7 and 11.8 % in Nicaragua, Honduras and Guatemala, respectively. The three main chronic conditions reported were hypertension, arthritis, and diabetes. Seeking care in the formal health system ranged from 73.4 % in Nicaragua to 83.1 % in Honduras, while full access to medicines varied from 71.6 % in Guatemala to 88.0 % in Honduras. The main associated factors of seeking care in the formal health system were geographic location, household head gender, Spanish literacy, patient age, perceived health status, perceived quality of public sector care, household economic level, and having health insurance. Seeking care in the formal health system was the main bivariate associated factor of obtaining full access to medicines (OR: 4.3 95 % CI 2.6 – 7.0). The odds of full access to medicines were significantly higher when the household head was older than 65 years, medicines were obtained for free, households had higher socioeconomic status, and health care was sought in the private sector.

**Conclusions:**

The nature of the health system plays an important role in access to medicines. Access is better when public facilities are available and function effectively, or when private sector care is affordable. Thus, understanding how people seek care in a given setting and strengthening key health system components will be important strategies to improve access to medicines, especially for populations at high risk of poor access.

## Background

Access to medicines is a component of access to health care and is considered by the World Health Organization (WHO) as fundamental human right [1]. Medicines are needed to achieve optimal health outcomes in a broad range of health conditions. When medicines are not used in a clinically effective way, they may precipitate adverse events or result in waste of scarce financial resources, leading to problems at individual, social, political, and economic level [1].

Ensuring access to medicines for people who need them is one of the major challenges in health policy, especially for non-communicable diseases (NCDs) [2, 3]. NCDs, such as cardiovascular disease, diabetes, cancer, and chronic respiratory diseases, are the leading causes of premature death and illness throughout the Americas. They account for some 4.5 million deaths each year in Latin America and the Caribbean, or 77 % of all deaths in the Region [2, 4, 5].

High blood pressure was one of the three leading risk factors for global disease burden, accounting for 9.4 million deaths, 7 · 0 % of global Disability-Adjusted Life Year (DALYs), in 2010.[6] Hypertension is one of the most important preventable causes of premature mortality worldwide [6] and in the American region, its prevalence is 35 % in the overall population [5].

The global prevalence of diabetes was estimated to be 9 % among adults aged 18 years and older in 2014. [7] In 2012, an estimated 1.1 million people died from diabetes. Almost 80 % of diabetes deaths occur in low- and middle-income countries. Almost half of diabetes deaths occur in people under the age of 70 years. In the American region, the number of people with diabetes was estimated at 62.8 million in 2011, and it is expected to reach the 91.1 million mark by 2030 [5]. Diabetes and its complications have a significant economic impact on individuals, families, health systems and countries [2, 4].

In the Latin America and Caribbean region, health expenditures are estimated to account for 33 % of all household expenditures and a large proportion of overall health expenditures is for medicines [5]. High out-of-pocket expenditures on medicines and high prices of medicines in the region represent important barriers to affordable access to care for NCDs [5].

Access to medicines is a complex phenomenon that is deeply intertwined with access to health care through the health system. Indeed, WHO recognizes medicines and technologies as one of the six health system building blocks [8]. In seeking approaches to improve access to medicines, it is important to consider a broad perspective on strengthening the health system [9]. Most health system strengthening interventions have ignored interconnections between system components. Consequently, population access to medicines has typically been addressed through fragmented, often vertical approaches [9].

A health system approach also implies considering the perspectives of patients, for whom the health system exists. Patients’ perceptions about access, quality of care, and affordability as well as their ability and willingness to pay for services drive utilization of the system, and strengthen or circumvent policies in place. Thus, analyses of access to care in different health systems need to recognize that health system components such as policy frameworks, structure and geographic location of services, health care financing, and medicines supply are interrelated [9, 10].

The study entitled *“Estudio del impacto de la exclusión de la atención de salud sobre el acceso a medicamentos en Honduras, Guatemala y Nicaragua”*, hereafter referred as the source study, used, linked household surveys in three Latin American countries to describe how different population groups obtain medicines and to assess the relationship between the exclusion from health care and access to medicines [11].

In this paper, we identify key factors related to access to medicines for treating chronic illness among persons who were reported to have at least one chronic condition.

## Methodology

This was a cross-sectional analytic study, based on data from a household survey conducted using methods developed by the World Health Organization (WHO) and adapted to study the exclusion from health care and access to medicines in Central America [11–13].

### Study settings

Household surveys were conducted in three Central American countries: Nicaragua, Honduras, and Guatemala.

The countries were selected according to the following criteria. (1) presented important challenges to achieve the Millennium Development Goals related to maternal mortality and access to health care; (2) had access to medicines as one component of the country’s technical cooperation agenda with PAHO/WHO; and (3) had a public health sector with limited resources, uncertain quality of services, and low coverage of basic public health services.

Selected profile aspects from the country health system are summarized in terms of social and demographic characteristics, health services, and health indicators in Table 1. The following sections include additional information about the three countries, specificities and highlights that could not fit in Table 1.

**Table 1:** General characteristics of study countries (Nicaragua, Honduras, and Guatemala)

	NIC	HON	GUT
Demographic^a^			
Population [Thousands]	5,142,098	7,536,952	12,728,111
Proportion of population 60 years and older [%]	3.2	5.2	3.6
Life expectancy at birth [Years]	70.8	73.0	68.7
Declares ethnic minority (%)	15	6	41
Socio-economic^a^			
Literacy rate [%]	74.1	78.0	71.0
Gross Domestic Product (GDP), per capita, international $ (PPP-adjusted) [US$]	3262	2665	4148
Gini index	40.1	57.7	55.1
Proportion of population below the international poverty line [%]	48.3	20.7	16.0
Unemployed proportion of the labor force [%]	12.2	3.8	1.8
Health services^b^			
Physicians’ ratio [10,000 hab.]	4.7	8.7	9.5
Number of outpatient care facilities [10,000 hab.]	2.2	2.8	1.2
Hospital beds ratio [per 1000 pop.]	0.9	1	0.5
Outpatient health care visits ratio [per 1000 pop.]	2154	1445	1071
Annual national health expenditure as a proportion of the GDP [%] [12]	3.7	2.0	1.4
Annual national health expenditure as a proportion of the GDP [%] (Private)	3.5	2.6	4.8
Health insurance coverage [%]	12.2	16.9	17.8
Health services coverage [%]	56.6	60.7	67.0
Health status (proportional mortality)^c^			
Cardiovascular diseases (CVD) (%)	25	31	14
Cancers (%)	13	13	11
Respiratory diseases (%)	4	4	2
Diabetes (%)	7	5	5
Others non communicable diseases (%)	20	16	15
Deaths estimated as caused by NCDs of all deaths (%)	69	69	47

^a^OPS, (Organização Pan-Americana da Saúde). Saúde nas Américas - Panorama Regional y Perfiles de país. Washington, D.C.: Organização Pan-Americana da Saúde; 2012

^b^PAHO, (Pan American Health Organization). Health Systems Profile in Nicaragua: Monitoring and Analyzing Health Systems Change/Reform. Washington, D. C.: PAHO2009.; PAHO, (Pan American Health Organization). Health System Profile Honduras, Central America: Monitoring and Analysis of Health Systems Change/Reform. Washington, D.C.:2009, PAHO, (Pan American Health Organization). Health systems profile of Guatemala. Washington, D.C2007

^c^WHO, (World Health Organization). Noncommunicable Diseases Country Profiles Geneva WHO, (World Health Organization) 2011

#### Nicaragua

Poverty and poor education were key social determinants of health status in Nicaragua [14]. In 2005, the estimated general poverty rate was 48.3 %, while an estimated 17.2 % of the population lived in conditions of extreme poverty. Only 52.4 % of the working age population was active in the job market in 2005; in 2003, secondary school completion rate was 45.2 and 25.9 % of the population is unable to read or write [14]. According to the 2005 census, 15 % of the population self-identified as belonging to an indigenous group or ethnic community [14].

Diseases of the circulatory system, injuries, and tumors accounted for the three leading causes of death and the morbidity and mortality associated with chronic diseases and injuries were increasing [14].

Nicaragua had no private insurance system with expanded coverage [14]. Nicaraguan Social Security Institute (INSS) provided the only health insurance, which is restricted to the formally employed population.

The Ministry of Health of Nicaragua (MINSA) has been the primary supplier of health services. Its service network targets primary and secondary care. The estimated care coverage rates of the country’s heath institutions were: MINSA (60 %); INSS (7.7 %, including beneficiaries and their family members); Ministry of Government and Military Health networks (8 %); and private institutions (4 %) [14].

Essential medicines were subsidized in the public sector, the government instituted free access to health care and medicines in the public sector in 2007 [11]. Availability of medicines was found to be 73.7 and 84.2 % in public and private health care sector respectively [11].

#### Honduras

In 2009, 80 % of households received only 36.8 % of the national income, while the wealthiest 20 % receive 63.2 % [15]. There were eight culturally distinct ethnic minorities (Lencas, Pech, Garifunas, Chortis, Tawahkas, Tolupanes/Xicaques, Miskitos, and an English-speaking black population), which accounted for 6 % of the population according to the 2001 census. The areas inhabited by the indigenous populations were some of the most deprived areas in the country, with limited access to basic services and infrastructure, subsistence economies, and environment problems [15].

The epidemiological profile of Honduras has been in transition. Infectious diseases, particularly conditions related to the respiratory and digestive systems, coexist with chronic degenerative conditions such as cancer and cardiovascular disease, which are more common in the adult population [15].

Hypertension and diabetes were the first and the sixth leading causes of specialized care in 2007, respectively. Although there was no complete database, chronic diseases constitute the leading causes of morbidity and mortality at the national level [15].

The health sector consisted of a public subsector made up of the Ministry of Health (SS), which played the steering and regulatory role in the sector, and the Honduran Social Security Institute (IHSS), which was responsible for collecting and managing fiscal resources and the required contributions made by workers and employers. Insured care was delivered by a mix of public and private sectors [15]. For-profit and nonprofit institutions formed the private subsector. Nine percent of the population was registered in the IHSS, 2.7 % has private insurance, and 88.3 % is covered by the SS [15].

The Honduras National Medicine Policy aimed to ensure adequate supply of quality medicines at the lowest possible price [11]. According to PAHO, by 2010 the Honduras government provided free medicines for the following health conditions: malaria, tuberculosis, sexually transmitted diseases, HIV/AIDS, and vaccines. Children under five, pregnant women and the elderly as well as people who have no ability to pay were also entitled to receive medicines from the basic medicine list free of charge in the public sector [11]. The Ministry of Industry regulated profit margins for importers and retailers of medicines; no other medicines price controls were in place. Availability of medicines was found to be 79.2 and 67.4 % in public and private health care sector respectively [11].

The IHSS purchased medicines through a centralized process that was separated from the SS. Procurement by the Ministry of Health was limited to medicines on the basic list, which was equivalent to a national essential medicines list. The IHSS limited its purchases to a separate list of medicines established by the institution [15]. Public health facilities charged a fixed fee per use, which covers the consultation, dispensing, laboratory, and clinic procedures [11].

#### Guatemala

More than half of the population lived in poverty and nearly 16 % in extreme poverty [16], but the GDP per capita was higher than $4000 US dollars, indicating a high level of socioeconomic disparities among populations groups. Overall, 41 % of the population were indigenous and more than half lived in rural areas.

Infectious and nutritional diseases were prominent in the country’s morbidity profile. Communicable diseases were the leading cause of mortality; however, deaths from cardiovascular diseases and tumors were increasing [16].

Health care in the public sub-sector was under the responsibility of a network of services from the Ministry of Public Health and Social Welfare [17] the Guatemala Social Security Institute (IGSS), the health services of the Ministries of Defense and Government, and the San Carlos University [16]. Medicines were provided free of charge to patients that cannot afford them and to the elderly, but only during inpatient hospitalizations, while children under five and pregnant women receive medicines free in both, hospitals and ambulatory care. However, except these two groups, the others did not have free access to continuous NCD outpatient treatment. Consultations and medicine dispensing required a fee per use in hospital and in ambulatory care [17]. Vaccines, contraceptives and medicines for specific diseases, such as malaria, tuberculosis, HIV/AIDS, sexual transmitted diseases were provided free of charge to the entire population due to their public health relevance [17]. Availability of medicines was found to be 64.7 and 88.2 % in public and private health care sector respectively [11].

### Sampling

Methods of the source study are detailed elsewhere [13]. Briefly, the sample was designed to estimate the proportions of persons excluded from health care and of those without access to medicines. A cluster sample was selected in three stages. In the first stage, 50 census tracts were selected, with probability proportional to size, based on the number of households; primary sampling units were stratified according to urban/rural location. In the second stage, 20 houses were selected in each census tract, anticipating a 25 % non-response rate. In the third stage, for multi-household units, one household was selected per unit using a table of random numbers [13].

The household survey evaluated access to health care and medicines at the household level for three categories of health conditions (acute, chronic, pregnancy) and also for respondents who reported poor health status [13]. Data were collected on one case of chronic illness per household. If the household reported more than one case, the oldest person experiencing an illness was selected [13].

### Study variables

We examined two key outcome measures: "seeking care in the formal health system" and “having full access to medicines” for a chronic condition (the latter defined as obtaining in the last month all medicines that were sought for the chronic condition reported among those who sought care in the formal health system).

The “formal health system” was considered here to include clinically trained personnel working in the health facilities of the Ministry of Health, the formal private sector (hospitals, private clinic and private physicians), or health facilities supported by the Social Security System [11].

The variable “economic level” was defined based on the number of goods in the household and the level of education of the head of the household, combined in a composite index [13]. This variable was categorized in three levels (A, B, and C) corresponding to high, middle, and low socioeconomic status, respectively.

The category “ethnic minority” was created for all three countries from respondent-reported categories, aggregating those who classified themselves as indigenous, black, or country-specific ethnic minorities [11, 13].

In this study we used the definition for urban and rural provided by each corresponding National Institute of statistics, responsible for the census information, as well as the area their map that classify the areas in urban or rural in the field [11].

### Statistical analysis

Data were analyzed with the statistical package SPSS® V.17 using descriptive statistics and multivariable logistic regression. First, bivariate analyses were performed to identify the variables associated with the two outcome variables. Results from initial analyses indicated that the variable "seeking care in the formal health system" was the most important associated factor of “having full access to medicines”. In addition, the three countries had distinct profiles concerning the most important determinants of seeking care and access to medicines for chronic diseases. Based on these preliminary analysis, we modeled the association (odds ratio) between the two primary outcomes and potential explanatory variables separately for each country.

All candidate explanatory variables were tested individually with both outcomes; multivariable logistic regression models included the explanatory variables found to be associated with the outcome variables with *p* <0.10 in bivariate analyses. If more than one candidate explanatory variables measured the same attribute (e.g. perception of geographic accessibility and rural/urban geographical location), the variable with the strongest association with the outcome variable was included.

For theoretical relevance of their relationships to both, access to health care and to medicines, the variables measuring economic and education level of the head of household were retained in all final multivariable models, along with all the other associated variables that had adjusted *p* < 0.10 in the multivariable models. Odds ratio (OR) estimates are presented in the paper with 95 % confidence intervals (CI).

Overall, the variables used in the analyses had a maximum of 5 % missing. Given this low percentage, we did not impute values for any missing variables, and cases with missing data were excluded from the analyses.

## Results

Overall, only about three-quarters of persons who reported a chronic condition sought care in the formal health system, ranging from 73.4 % in Nicaragua to 83.1 % in Honduras (Table 2). Of those who sought care in the formal health system, almost all were successful in obtaining health care and they were more likely to have *“full access to medicines”* for their chronic illness, (*p* <0.01) Nicaragua (OR: 9.4 95 % CI 5.0 – 17.6) Honduras (OR: 6.1 95 % CI 3.0 12.4) and Guatemala (OR: 13.3 3.8 – 47.2). Seeking care in the formal health system was the main bivariate associated factor of obtaining full access to medicines (OR: 4.3 95 % CI 2.6 – 7.0).

**Table 2:** Health seeking behavior and access to medicines by country

Country	NIC	HON	GUT
Number of households with a chronic condition	450	267	110
Sought care in the formal health system (%)	73.4^◊◊◊^	83.1^◊◊◊^	73.8^◊◊◊^
Obtained health care in the health system	72.1	81.4	68.1
Sought care in the public sector (%)	68.3	60.2^◊◊^	47.6^◊^
All medicines prescribed by a doctor (%)	88.5	88.9	89.2
Sought medicines (%)	77.5	80.1	59.9
Full access to medicines	81.3	88.0	71.6
All medicines obtained in the public sector (%)	35.6	32.1^◊◊^	10.8
All medicines obtained in the private sector (%)	47.2	56.1	23.3
At least one medicine obtained in the private sector (%)	56.1	66.9^◊◊^	33.3
Obtained medicines for free (%)	49.8^◊◊^	33.7^◊◊^	29.2

Full access to medicines^◊◊◊^ *p* <0.01, ^◊◊^ *p* <0.05, ^◊^ *p* <0.1

A majority of respondents who sought care did so in the public sector in Nicaragua (68.3 %) and Honduras (60.2 %), while less than half (47.6 %) did so in Guatemala, being this aspect associated with *“full access to medicines”* in Honduras (*p* < 0.05) and Guatemala (*p* < 0.1). Most medicines were prescribed by doctors in all three settings.

Among those reporting a chronic condition, “*full access to medicines”* varied from 71.6 % in Guatemala to 88.0 % in Honduras. In all countries, the majority of medicines were obtained in the private sector and less than half of persons who obtained medicines received all of them free of charge. *“full access to medicines”* was associated to *“All medicines obtained in the public sector”* and *“at least one medicines obtained in the private sector”* in Honduras (*p* < 0.05) and *“Obtained medicines for free”* was associated in both Nicaragua and Honduras (*p* < 0.05).

Overall, 29.8 % (827) of 2761 households reported in at least one chronic condition. In Nicaragua, Honduras and Guatemala this percentage was 47.0, 30.7 and 11.8 % of households, respectively (Table 3).

**Table 3:** Characteristics of households (HH) with chronic conditions and bivariate associations with the outcome variables by country, 2010

Country	NIC	HON	GUT
Total number of HH	957	869	935
Number (%) of HH reporting one or more chronic diseases	450	267	110
Number of persons with a chronic disease per HH [mean(SE)]	1.2 (0.5)	1.2 (0.5)	1.1 (0.5)
HH located less than 30 min for public health facility (%)	71.3*^, ◊◊^	71.0	75.6
HH located less than 30 min for private health facility (%)	50.1^◊◊^	52.8^**,◊◊◊^	47.3
HH located in urban region (%)	62.9***	51.4	58.9**^,◊◊◊^
HH with fewer than 3 persons per room (%) (overcrowding)	84.6**	90.7	96.6
Economic level	*	**^,◊◊^	**^,◊◊◊^
A (wealthy)	25.5	29.9	16.7
B (middle)	44.7	47.1	53.4
C (poor)	29.8	23.0	29.9
HH head is female (%)	61.1**	32.8	35.4
HH head can read and write in Spanish (%)	79.7^◊◊^	75.5	85.3
Age of the head of household [mean (SE)]	52.4 (15)	54.7 (15)	51.2 (15)
HH head not an ethnic minority (%)	84.9	84.9^◊◊^	93.6^◊◊^
HH head educational level	**		***^,◊^
None or less than primary school (%)	53.4	57.0	37.4
Primary school (%)	30.9	24.1	27.7
Secondary school and more (%)	15.7	18.9	34.9
Head of the household is employed (%)	25.9	27.1	25.0^◊◊^
Head of the household is retired (%)	12.4***^,◊◊^	13.5	10.3^◊^
HH health insurance (%)	21.8*	14.8	22.4***^,◊◊^
HH health insurance that cover all medicines (%)	8.6^◊◊^	8.6	14.3^◊◊^
Perceptions			
Geographic location of public health facility is good (%)	61.4***^,◊◊^	66.1	60.6^◊◊^
Working time of public health facility is good (%)	57.8	69.9	64.0
Working time of the pharmacy at public health facility is good (%)	56.2	68.9	69.2
Quality of the health care at public health facility is good (%)	51.5***	64.5	45.1*
Quality of the medicines at public health facility is good (%)	61.1*	68.1	56.6

Sought health care in the health system ***p <0.01, **p <0.05, *p <0.1

Full access to medicines ^◊◊◊^ *p* <0.01, ^◊◊^ *p* <0.05, ^◊^ *p* <0.1

The general socio-demographic profile was similar across countries. Few households were overcrowded (defined as three or more persons per room), ranging from 3.4 % in Guatemala to 15.4 % in Nicaragua. In all countries, households reporting a chronic health condition were located primarily in urban areas. Overall, more than 71.0 % of households had a public health facility located less than 30 min away, ranging from 71.0 % in Honduras to 75.6 % in Nicaragua. Geographic accessibility of private health facilities located within 30 min varied from 47.3 % in Guatemala to 52.8 % in Honduras.

Nicaragua had a much higher proportion of female heads of household (61.1 %) compared to Honduras (32.8 %) and Guatemala (35.4 %). Overall, few households identified the female head of household as being a member of an ethnic minority, ranging from 6.4 % in Guatemala to 15.1 % in Nicaragua and Honduras. In Guatemala, 34.9 % of household heads had secondary school education or higher, compared to 15.7 and 18.9 % in Nicaragua and Honduras, respectively.

Respondents in all three countries reported poor health insurance coverage, with only 14.8 % (Honduras) to 22.4 % (Guatemala) of households covered and less than 15 % of households in any country having health insurance with medicines coverage (8.6 % in Honduras and Nicaragua to 14.3 % in Guatemala).

Around 60 % of households had a positive assessment of the geographic location, working time, quality of care, and medicines quality in nearby public health facilities. This proportion was somewhat higher in Honduras than in Nicaragua or Guatemala.

In Honduras and Guatemala, the majority of people reporting a chronic condition was female, while in Nicaragua the majority was male (Table 4). More than 75 % of the chronic conditions reported in the survey were in persons with age 41 and greater, which is consistent with the epidemiology of chronic illness and also with the field protocol to collect information about the oldest person with a chronic condition in each household.

**Table 4:** Characteristics of individuals with chronic diseases and bivariate association with the outcome variables by country, 2010

Country	Nicaragua	Honduras	Guatemala
Number of households that report a chronic condition	450	267	110
Person with a chronic condition			
Female (%)	37.2	64.5	57.3
Age [mean (SD]	52.2 (19)	51.5 (22)	50.8 (19)
Age (%)	^◊^	^◊◊^	
Under 15	4.4	8.7	6.6
16 – 40 years	19.5	18.0	18.1
41 – 64 years	49.9	43.5	54.5
65 and over	26.2^◊^	29.8	20.8
Not an ethnic minority (%)	85.8	83.7	90.1^◊◊^
Reads/writes in Spanish (%)	78.5	73.9**	74.9^◊^
Relationship with the head of household			**
Head of the household or spouse (%)	80.8	77.7	82.6
Son/ daughter/ grandchild / stepchild (%)	7.1	12.3	6.4
Other relative (%)	11.5	10.0	11.0
Not related (%)	.5	0	0
Difficulty in carrying out normal daily activities (%)		*	*
None (no difficulty in carrying our normal activities)	18.9	22.1	16.4
Low	28.1	24.5	26.8
Medium	29.8	24.6	27.3
High	14.4	19.5	24.2
Extreme (cannot carry out normal activities)	8.5	9.3	2.8
Chronic condition reported (%)			
Hypertension	47.1	45.7	15.5
Arthritis	39.3	21.7	13.6
Diabetes	18.0	18.4	27.3
Cardiovascular diseases	14.4	13.5	10.0
Asthma	11.6	13.5	5.5
Stomach diseases	10.7	11.6	12.7
High cholesterol	6.9	10.1	4.5
Other	14.2	18.4	13.6
Health status self-evaluated as good (%)	53.8	43.3**	41.0
Health insurance (person with chronic disease only) (%)	17.4**	11.0	13.3*^,◊◊◊^
Health insurance covers medicines (person with chronic disease only) (%)	14.4**^,◊◊^	11.0	11.2*^,◊◊◊^

Sought health care in the health System ***p <0.01, **p <0.05, *p <0.1

Full access to medicines ^◊◊◊^ *p* <0.01, ^◊◊^ *p* <0.05, ^◊^ *p* <0.1

Considering the three countries combined, 45 % of the persons with a chronic condition reported medium or high levels of difficulty in carrying out normal activities, while 8 % reported extreme difficulty.

The three main chronic conditions reported were hypertension, arthritis, and diabetes in all countries. However, the percentage of individuals reporting each condition varied across countries. Almost half of the chronically ill individuals in Nicaragua (47.1 %) and Honduras (45.7 %) reported having hypertension, compared with a much lower percentage in Guatemala (15.5 %). Arthritis, as well, was more reported in Nicaragua (39.3 %) and Honduras (21.7 %) then in Guatemala (13.6 %). Conversely, proportions of chronically ill individuals reporting diabetes were higher in Guatemala (27.3 %) than in Nicaragua (18.0 %) or Honduras (18.4 %) (Table 4). Consistent with these conditions, the three main categories of medicines reported were antihypertensive, anti-diabetic, and anti-inflammatory medications.

### Care seeking

In Nicaragua, bivariate analyses found that seeking care in the formal health system was associated with the following variables related to the household: higher educational level, higher economic level, urban region, vicinity to health facilities, female head of household, retired head of the household, favorable perceptions about geographic location and quality of care in the nearest public health facility, household wealth attributes (electric power, own bathroom), health insurance (head of the household and person with chronic condition), and having health insurance that covers medicines for the individual with chronic condition (significant explanatory factors are denoted in Tables 3 and 4). In the final multivariable model, the significant variables associated with “*seeking care in the formal health system”* were household located <30 min to a public health care facility (OR: 3.0 95 % CI 1.6 – 5.8), female household head (OR: 1.9 95 % CI 1.3 – 2.8), and quality of care in public health facilities perceived as good (OR: 2.6 95 % CI 1.5 – 4.5) (Table 5).

**Table 5:** Predictors from multivariable logistic regression models of seeking health care for a chronic disease in the formal health system in Nicaragua, Honduras and Guatemala, 2010

Nicaragua			
Economic and demographic variables	OR	CI	*p*-value
Economic level			
A (wealthy)	1.0	0.4 – 2.6	1.00
B (middle)	1.5	0.9 – 2.7	0.15
C (poor)	1.0	–	
HH head educational level			
Secondary school+	2.1	0.9 – 4.6	0.07
Primary school	1.1	0.7 – 1.7	0.69
None or < primary school	1.0	–	
Other individual and household variables			
Household located <30 min to the public health care facility	3.0	1.6 – 5.8	0.00
Female HH head	1.9	1.3 – 2.8	0.00
Quality of care in the public health facility perceived as good	2.6	1.5 – 4.5	0.00
Honduras			
Economic and demographic variables	OR	CI	*p*-value
Economic level			
A (wealthy)	2.8	0.9 – 9.2	0.08
B (middle)	1.9	0.9 – 4.1	0.10
C (poor)	1.0	–	–
HH head educational level			
Secondary school+	0.9	0.2 – 3.6	0.89
Primary school	1.6	0.5 – 4.6	0.41
None or < primary school	1.0	–	
Other individual and household variables			
Literacy in Spanish (individual with chronic condition)	2.0	1.0 – 4.4	0.07
Health status perception evaluated as fair or bad	2.7	1.2 – 6.5	0.02
Guatemala			
Economic and demographic variables	OR	CI	*p*-value
Economic level			
A (wealthy)	0.3	0.1 – 1.9	0.11
B (middle)	1.1	0.4 – 3.4	0.87
C (poor)	1.0	–	
HH head educational level			
Secondary school+	2.0	0.4 – 9.9	0.40
Primary school	0.3	0.1 – 0.9	0.03
None or < primary school	1.0	–	
Other individual and household variables			
Health insurance (HH head)	10.5	1.0 – 121.3	0.05
Quality of care in the public health facility perceived as good	2.8	1.0 – 10.1	0.08

In Honduras, households with a higher odds of seeking care for a chronic illness in the formal health system were those located at <30 min to a private health care facility, with high economic level, individual with chronic condition able to read and write in Spanish, where the chronically ill person reported high or extreme difficulties in performing daily activities, and poor perceived health status (Tables 3 and 4). In the final multivariable model, the significant associated variables with seeking care in the formal health system were households with higher economic level (OR: 2.8 95 % CI 0.9 – 9.2), individuals with chronic condition that has literacy in Spanish (OR: 2.0 95 % CI 1.0 – 4.4), and health status perception evaluated as fair or bad (OR: 2.7 95 % CI 1.2 – 6.5) (Table 5).

In Guatemala, household location in an urban area, higher economic level, higher educational level, having health insurance, positive perception of the quality of care in the nearest public health facility, and chronic ill individual reporting difficulty in carrying out normal activities were the factors associated with seeking care for chronic illness (Tables 3 and 4). In multivariable models, the odds of seeking care were higher for households insurance (OR: 10.5 95 % CI 1.0 – 121.3) and for those that had positive perceptions about the quality of care in the public health care facility (OR: 2.8 95 % CI 1.0 – 10.1).

### Access to medicines

The most important associated factor of obtaining full access to medicines in bivariate analyses was seeking care in the formal health system (Table 2).

In Nicaragua, bivariate correlates of full access to medicines included proximity to public and private health facilities, literacy in Spanish, having a retired household head, medicines insurance coverage, age over 65, and paying for medicines. Having a household head older than 65 years (OR: 5.3 95 % CI 1.1 – 25.4) and receiving all medicines free of charge (OR: 2.7 95 % CI 1.0 – 7.1) were the significant associated variables in the final multivariable model (Table 6).

**Table 6:** Predictors from multivariable logistic regression models of obtaining full access to medicines for a chronic disease among those seeking health care in the formal sector, Nicaragua, Honduras and Guatemala, 2010

Nicaragua			
Economic and demographic variables	OR	CI	*p*-value
Household level			
Economic level			
A (wealthy)	1.0	0.1 – 6.1	0.99
B (middle)	1.1	0.3 – 3.4	0.89
C (poor)	1.0	–	
HH head educational level			
Secondary school+	1.2	0.4 – 7.0	0.81
Primary school	1.1	0.4 – 3.7	0.88
None or < primary school	1.0	–	
Other individual and household variables			
65 years and over	5.3	1.1 – 25.4	0.04
Had all medicines for free	2.7	1.0 – 7.1	0.05
Honduras			
Economic and demographic variables	OR	CI	*p*-value
Household level			
Economic level			
A (wealthy)	5.1	2.1– 12.3	0.00
B (middle)	1.2	0.5 – 3.3	0.72
C (poor)	1.0	–	
HH head educational level			
Secondary school+	0.4	0.2 – 1.0	0.03
Primary school	3.5	0.7 – 17.2	0.13
None or < primary school	1.0	–	
Other individual and household variables			
Obtained at least one medicine in the private sector	2.4	1.0 – 6.3	0.06
Guatemala			
Economic and demographic variables	OR	CI	*p*-value
Household level			
Economic level			
A (wealthy)	3.7	0.4 – 39.3	0.27
B (middle)	2.0	0.6 – 6.8	0.27
C (poor)	1.0	–	
HH head educational level			
Secondary school+	3.4	1.0 – 13.2	0.06
Primary school	0.8	0.2 – 2.6	0.75
None or < primary school	1.0	–	
Other individual and household variables			
Sought health care in the private sector in a chronic condition	3.1	1.1 – 8.8	0.03

In Honduras geographic proximity to a private health facility, household economic level, head of the household being an ethnic minority, age of the individual with chronic condition, receiving free medicines, seeking care in the private sector, and obtaining medicines in the private sector were the main bivariate associated factors of full access to medicines. In the final multivariable model, household economic level (OR: 5.1 95 % CI 2.1 – 12.3) and obtaining at least one medicine in the private sector (OR: 2.4 95 % CI 1.0 – 6.3) were the significant associated variables of full access to medicines (Table 6).

In Guatemala urban location of the household, higher economic level, not being a member of an ethnic minority, higher educational level, being employed or retired, having health insurance, having positive perception about the geographic accessibility of a public health facility, and seeking care in the private sector were the main associated variables of having full access to medicines. In multivariable models, the odds of having full access to medicines were significantly higher for those who sought health care in the private sector (OR: 3.1 95 % CI 1.1 – 8.8) and who had a higher educational level (OR: 3.4 95 % CI 1.0 – 13.2 ) (Table 6).

In general, the main reasons reported for not obtaining full access to medicines were similar in all countries (data not shown). These included “do not have money” (66.9 %), “medicines were not available at the pharmacy or health care facility” (18.9 %) and “prices of medicines are high” (15.6 %).

## Discussion

Access to medicines is a complex phenomenon, embedded in an equally complex health system [7]. The main associated factor of obtaining access to medicines for treating chronic illness in these three Latin America countries was seeking care in the formal health system. Despite the informal availability of medicines in these settings, most people use the formal health care system to seek care for NCDs. This indicates that promoting an accessible and functioning health system will be a sound strategy to enhance access to medicines for individuals with chronic disease.

The percentages of households reporting a chronic illness in these surveys were lower than those previously found in Brazil (43.8 %) [18], Oman (44.4 %) [19] and Philippines (39.0 %) [20] in similar surveys, but higher than rates found in surveys in Ghana (16 %) [21] and Nigeria [22]. The countries in the current study have a relatively young population [5], which may explain the low percentages of respondents reporting a household member with a chronic disease. Notably, the survey required patients to have a prior diagnosis of chronic illness by a trained health worker as a criterion for entry to the study. Low diagnostic capacity in these health systems could have led to an underestimation of the number of cases of chronic disease. The most common diseases reported (hypertension, arthritis, diabetes) were similar to those found in other studies [18–22] and also to those reported in previous studies of health in the Americas [5].

The health seeking behavior in a chronic condition is quite different from in acute condition. This study results pointed out that overall more than 70 % of the individuals with chronic condition sough care in the formal health system. The literature points out that in an acute condition, this percentage is lower, less than 50 % [12] and 30 % [23] sought care for acute conditions, being the perception of severity one of the associated factors [12, 23].

In these three settings, most people who sought care were able to obtain full access to medicines for chronic conditions. Nevertheless, the percentages of individuals who reported full access to medicines were lower than rates found in other studies in the Americas (89.6 % in Brazil [24] and 97 % in [25], but similar to rates in two states in Brazil (81 % among the elderly [26] and Colombia (75.1 %) [27].

Spending for health care is an important component of overall household spending in many countries, and having to pay for health care is a key source of economic burden and impoverishment for many of the poorest members of the population [28–31]. Demand for medicines tends to be particularly inelastic and people are often willing to sell assets or ask for credit to afford them [32, 33]. Treating chronic illness can present a continuous drain on financial resources, with medicines expenditures constituting a substantial proportion of overall health spending [2, 4, 34–36]. Social protection for health care through a well-functioning public sector or widespread insurance coverage is lacking for a significant proportion of the population in Latin America and the Caribbean [13, 28, 37]; indeed, affordability of care proved to be an important problem in the countries addressed in this study. Chronically ill people are especially vulnerable since they require continuous care and impaired health status may also diminish their ability to work, leading to a vicious cycle of impoverishment and poor health.

Medicines were available free of charge to the elderly population in Nicaragua and Honduras, yet many elderly people were found to have paid for their medicines. This may point to the existence of other barriers to accessing services, including low education and health literacy, poor geographic and functional accessibility, inefficiencies in the public sector medicines supply system, and negative perceptions about public sector service quality. Indeed, availability of medicines was found to be low in the three countries, always below or around 80 %, both in public and in private sector. The economic level of households was a significant determinant of access to medication except in Nicaragua, which was the only one of the three countries to guarantee free provision of medicines to the general population. Nevertheless, even there, less than half of respondents received medicines free of charge.

Geographical barriers (distance and transportation difficulties), economic factors (cost of consultation and medicines), and cultural barriers (Spanish illiteracy, beliefs about illnesses and treatments) have been identified as barriers to access to health services in other studies in Central America [13, 38, 39].

A high percentage of individuals sought care in the formal health sector in all three countries, but about one in five who reported having a chronic illness did not do so. Chronic diseases typically require continuous access to health care and medicines to prevent poor health outcomes. The primary reasons reported for not seeking care tended to be financial. However, other aspects of the health system such as a low or inadequate geographical coverage, poor quality of health services (real or perceived), and inefficient patterns of health seeking behavior may also explain the failure to seek care. Health insurance coverage was low in all three countries; this has been identified in the literature as a major barrier to access to health care and medicines [28, 40–43].

Care-seeking behavior is strongly related to individuals’ overall perceptions about their health status, severity of illnesses, and the consequences of not treating them. In addition, care-seeking may reflect beliefs about the quality of the health care services or ability to overcome perceived barriers to access [40, 44–46]. Understanding more about the reasons why people do not seek care or seek care in settings where treatment is not free are important topics for future research.

The number of people who identified themselves as an ethnic minority was lower than expected, especially in Guatemala; this may have been due to underrepresentation of areas with high concentrations of ethnic minorities in these studies due to sample design, or to underreporting of belonging to an ethnic group. Nevertheless, persons with chronic illness who identified themselves as ethnic minorities were less likely in bivariate analyses to seek care and to receive medicines, a finding which has been mentioned in the literature [13, 38, 39, 47]. However, ethnicity was not a significant associated factor in the final multivariable model due to its correlation with stronger associated factors such as household income and geographic distance from health facilities. Nevertheless, it is important to understand how being in an ethnic minority affects seeking care and obtaining access to medicines, since this represents an important equity issue.

The reliability and efficiency of the public sector may have substantial impact as on availability and quality of medicines [48]. Previous studies have shown that both availability and cost of medicines are higher in the private sector regardless of country [5, 49, 50]. More than half of medicines in these surveys were obtained in the private sector. The cost of treating chronic illness in the region is generally more than one day of work at a minimum wage [5, 49, 50]. Low public sector availability of free medicines combined with high private sector prices may constitute a substantial barrier in access to medicines in these countries, especially in light of low coverage of health insurance.

Our findings indicate the need of more harmonization between health and pharmaceutical policies. Social protection mechanisms, including insurance coverage of medicines and provision of free medicines in the public sector, are strategies to improve access. In addition, other barriers to medicines access such as geographical accessibility and perceived quality of care also call for public policy interventions, combined with educational campaigns and partnerships with the civil society.

Our findings suggest that optimal policy approaches to improve access to medicines may differ by country health system context. Taking in account the health systems perspective was useful in shedding light on some key linkages. As example, In Nicaragua, despite the highest unemployment and proportion of people below poverty line among the three countries, had the highest ratio of public health expenditure and also free access to medicines for the overall population. In this country, most health care was provided in public health facilities and the main determinants for seeking health care were location and perceived quality of services. The free provision of medicines to the general population was an important determinant for full access to medicines. In Honduras, where a flat copayment rate was in place for health care and medicines, perceived need and economic status were important determinants for seeking health care and private pharmacies were the main source for medicines.

In Guatemala, with the highest ratio of physicians per capita among the three countries, had high private sector utilization. Indeed, health insurance coverage was an important determinant for seeking health care, and economic status and level of education were associated with access to medicines.

As household survey linked to secondary data, the current study may have limited external validity. The study was not designed to infer causality, since the exposures and outcomes were simultaneously assessed and no temporal relationships were assessed [51, 52]. When considering population characteristics such as gender or ethnicity. The temporal nature of the exposure-outcome association is more plausible. However, for factors such as economic level, reverse causality is possible, e.g., if limited access to medicines leads to reduced economic circumstances. Access to medicines is a complex concept and there may be diverse pathways through which associated factors operate.

Limitations also include the relatively small sample size, the inclusion of specific geographic areas in each country, and underrepresentation of ethnic minorities, especially in Guatemala. Additionally some country-specific results were not as expected, such as the association between education and access to medicines in Honduras, where households with more highly educated heads were less likely to have access to medicines. One possible hypothesis is that public programs might increase access to medicines for the less well-educated part of the population that accesses care in public facilities.

We used a health system perspective in exploring the relationships among different health system factors on household access to medicines, but we missed information from other stakeholders such as health providers or policy makers. Nevertheless, we believe this is an important approach, since end users are the reason for the existence of health systems. Despite possible inconsistencies in the information they provide, the perceptions of household respondents provide a window on how they interact with the health system.

Despite these limitations, the study was successful in identifying key barriers in access to medicines for household members with a diagnosed chronic condition in three Central America countries.

## Conclusion

These results increase our understanding of the most significant barriers to access to the formal health sector and to medicines in three different settings in Latin America, and they suggest policies and strategies that could increase access to medicines and equity. A health system perspective was helpful in understanding findings. Seeking care in the formal health system was the main determinant of access to medicines for chronic conditions. Thus, strengthening health systems and improving community perceptions about quality of care will be important strategies to improve access to medicines. This is especially true for people at high risk, such as those living in rural areas, women, ethnic minorities, and those with low education or economic disadvantage. Among the poor in these surveys, most people paid out of pocket for their medicines. Expanding insurance coverage and other types of social protection will assure greater access to medicines among those who cannot currently afford them.
